# The Evaluation of Hydroxyethyl Starch (6% HES 130/0.4) Solution’s Potential Preventive Effects on Coagulation Status in Women with Gynecologic Malignancies Using Rotation Thromboelastography

**DOI:** 10.4274/tjh.2013.0003

**Published:** 2014-09-05

**Authors:** Meltem Olga Akay, Ayten Bilir, Tufan Öge, Gökhan Kuş, Fezan Şahin Mutlu

**Affiliations:** 1 Eskişehir Osmangazi University Faculty of Medicine, Department of Hematology, Eskişehir, Turkey; 2 Eskişehir Osmangazi University Faculty of Medicine, Department of Anesthesiology and Reanimation, Eskişehir, Turkey; 3 Eskişehir Osmangazi University Faculty of Medicine, Department of Obstetrics and Gynecology, Eskişehir, Turkey; 4 Eskişehir Osmangazi University Faculty of Medicine, Department of Biostatistics, Eskişehir, Turkey

**Keywords:** Blood Coagulation, Colloids, Genital neoplasms, Thromboelastography

## Abstract

**Objective:** The aim of this study was to determine the effects of in vitro hemodilution with 6% hydroxyethyl starch (HES) 130/0.4 solution on the coagulation status of women with gynecologic malignancies by using rotation thromboelastogram (ROTEM®).

**Materials and Methods:** Twenty-two patients with gynecological tumors scheduled for anesthesia were enrolled. Blood samples were diluted by 20% with 6% HES (130/0.4) solution.

**Results:** In the INTEM assay, clotting time (CT) (p<0.01) and clot formation time (CFT) (p<0.001) were significantly increased and maximum maximum clot formation (MCF) (p< 0.001) was significantly decreased in HES hemodilution compared with the undiluted control samples. In the EXTEM assay, there was a similar significant increase in increase in CFT (p<0.01) and a decrease in maximum a decrease in MCF (p<0.01) in HES hemodilution when compared with control samples.

**Conclusion:** HES 130/0.4 solution causes significant hypocoagulable changes in the thromboelastographic profile of gynecologic cancer patients in vitro.

## OZET

**Amaç:** Bu çalışmanın amacı, jinekolojik maligniteli kadınlarda %6 hidroksietil nişasta (HES) 130/0,4 solüsyonu ile hemodilüsyonun koagülasyon durumu üzerine in vitro etkilerinin rotasyonel trombelastogram (ROTEM®) kullanarak belirlenmesidir.

**Gereç ve Yöntemler:** Anestezi programına alınan jinekolojik tümörlü 22 hasta dahil edildi. Kan örnekleri %6 HES (130/0,4) solüsyonu ile %20 dilue edildi.

**Bulgular:** INTEM analizinde, HES hemodilüsyonunda, dilüsyon yapılmayan kontrol örneklerine göre pıhtılaşma zamanı (CT) (p<0.01) ve pıhtı oluşma zamanı (CFT) (p<0,001) anlamlı uzamış ve maksimum pıhtı sertliği (MCF) (p<0,001) anlamlı kısalmış idi. EXTEM analizinde, HES hemodilüsyonunda, dilüsyon yapılmayan kontrol örneklerine göre benzer olarak CFT değerinde anlamlı uzama (p<0,01) ve MCF değerinde anlamlı kısalma (p<0,01) mevcut idi.

**Sonuç:** HES 130/0,4 solüsyonu jinelolojik kanserli hastaların trombelastografik profilinde in vitro önemli hipokoagulabl değişikliklere yol açmaktadır.

## INTRODUCTION

Venous thromboembolism (VTE) is a serious and frequent problem in gynecologic cancer patients. Malignancy is associated with a baseline hypercoagulable state due to many factors including release of inflammatory cytokines, activation of the clotting system, expression of hemostatic proteins on tumor cells, inhibition of natural anticoagulants, and impaired fibrinolysis [1]. On the other hand, several risk factors related to the patient, cancer type, and therapeutic interventions such as central venous catheters, perioperative transfusion, systemic treatments including chemotherapy and hormone therapy, and surgery are associated with an increased risk of VTE in cancer patients [2]. Surgery in gynecologic cancer, as an important contributing factor to hypercoagulable states, shows several differences from general surgery. Patients often require intrapelvic procedures, such as lymph node dissection and excision of peritoneal metastases, which significantly increase the incidence of VTE in gynecologic cancer patients [3]. The incidence of asymptomatic deep VTE based on objective diagnostic screening is given as 15%-40% among patients undergoing major gynecologic surgery without preventive measures by the American College of Chest Physicians’ guidelines [4].

Intravascular volume deficits often occur during major abdominal surgery and adequate restoration of intravascular volume is an important therapeutic maneuver in managing the surgical patient care. Synthetic colloid solutions like gelatins and hydroxyethyl starch (HES) preparations are frequently used as an alternative for blood loss replacement to restore intravascular volume and avoid the risk associated with transfusion of allogenic blood products [5,6]. Gelatins have little negative influence on the coagulation process, whereas HES, especially if administered in large volumes, can impair coagulopathy because of reduced release of factor VIII/von Willebrand Factor, impaired platelet function, and hemodilution. Their compromising effects on blood coagulation are mainly dependent on their molecular weight (MW) and degree of substitution (DS). Recently developed 6% HES 130/0.4 solutions impair coagulation to a lesser degree, because of lower mean MW and lower DS [7]. From a theoretical point of view, HES-related coagulopathy can attenuate the hypercoagulable states. In this context, we hypothesized that besides the advantage of avoiding transfusion-associated risks, HES may have potential beneficial effects on the hypercoagulable state of gynecologic cancer surgery when used for the restoration of intravascular volume deficits.

This study was designed to assess the influence of 6% HES 130/0.4 preparation on hemostasis by using modified rotation thromboelastogram in women with gynecologic malignancies in vitro.

## MATERIALS AND METHODS

After obtaining approval from our institute’s ethics committee on 18 May 2012 and patients’ informed consent, 22 patients with gynecological cancer (13 endometrial and 9 ovarian) scheduled for anesthesia were enrolled in this study between 18 May and 30 June 2012. Patients with a history of hematological or coagulation disorders, those taking anticoagulant therapy, and those with renal or liver dysfunction were excluded.

Blood was withdrawn from each volunteer using a 19-gauge needle under minimum stasis. All blood samples were collected using a 2-syringe technique. After discarding the initial 2 mL of blood to prevent tissue thromboplastin contamination, samples for thromboelastography (TEG) analysis were drawn into 4.5-mL vacutainers (Becton Dickinson) containing 3.2% trisodium citrate with a citrate/blood ratio of 1:9. After harvesting the sample, the blood was diluted by 20% using 6% HES 130/0.4 (Voluven®, Fresenius Kabi, Bad Hamburg, Germany); 20% hemodilution corresponds to a volume of HES infusion that simulates the recommended blood replacement volume used clinically in the perioperative period. Undiluted whole blood acted as a control. Blood coagulation of HES dilution and the undiluted control was assessed using TEG. 

**Thromboelastography**

TEG analysis was performed with the ROTEM® Coagulation Analyzer (Pentapharm, Munich, Germany). After incubating the test solution at 37 °C for 2 min, 300 µL of citrated blood was recalcified with 20 µL of 0.2 mol/L CaCl2 (star-TEM®, Pentapharm) and activation of coagulation was performed with different agents. In INTEM, coagulation is activated with 20 µL of contact activator (partial thromboplastin-phospholipid from rabbit brain extract and ellagic acid, in-TEM®, Pentapharm). In EXTEM, coagulation is activated by 20 µL of tissue factor (tissue thromboplastin from rabbit brain extract, ex-TEM®, Pentapharm).

The method and the parameters of ROTEM® have been described in detail previously [8]. All TEG samples were analyzed within 30-90 min of blood collection by the same investigator. The integrated computer of the device calculated the following standard variables: clotting time (CT), clot formation time (CFT), and maximum clot formation (MCF). The CT is the time from start of the measurement until initiation of clotting. CT is influenced by activities of coagulation factors. CFT is the time from initiation of clotting until a clot firmness of 20 mm is detected. CFT is influenced by activities of coagulation factors, platelet count/function, thrombin formation, fibrin precipitation, fibrinogen, and hematocrit. MCF represents the firmness of the clot. It is affected by fibrin and fibrinogen concentration, platelet count/function, thrombin concentration, factor XIII, and hematocrit.

**Statistics**

Statistical analysis was carried out using IBM SPSS Statistics 20. Normally distributed continuous dependent variables were analyzed using the paired t-test and are presented as mean and standard deviation. Nonnormally distributed variables were compared with the Wilcoxon test for 2 dependent groups and are presented as median (25th to 75th percentile). A p-value of less than 0.05 (p<0.05) was accepted as significant. 

## RESULTS

Results of ROTEM® parameters are presented in Table 1. In the INTEM assay, CT (p<0.01) and CFT (p<0.001) were significantly increased and MCF (p<0.001) was significantly decreased in HES hemodilution compared with undiluted control samples. In the EXTEM assay, there was a similar significant increase in CFT (p<0.01) and a decrease in MCF (p<0.01) in HES hemodilution when compared with control samples. As anticipated, the ROTEM® parameters of 20% HES-diluted whole-blood samples of patients with gynecological tumors suggested a hypocoagulable state when compared with the baseline parameters. This hypocoagulability was diagnosed readily by the presence of decelerated clot formation, as evidenced by increasing of CT and CFT, and a decrease of the clot strength, as evidenced by shortening of MCF.

## DISCUSSION

The results of the present study confirm that 6% HES 130/0.4 does influence the coagulation process in vitro. ROTEM® parameters of HES-diluted whole-blood samples of patients with gynecological tumors suggested a hypocoagulable state when compared with the control parameters.

Cancer patients, and especially those undergoing surgery for cancer, are at extremely high risk for VTE, even with appropriate thromboprophylaxis [2]. The risk of VTE in cancer patients undergoing surgery is 3 to 5 times greater than that in surgical patients without cancer and this status is directly related with postoperative morbidity, mortality, and higher health care costs [9,10]. Gynecologic cancer surgery shows several differences from general surgery. Patients often require intrapelvic procedures, such as lymph node dissection and excision of peritoneal metastases, and it is likely that VTE will occur at a high incidence in these patients [3].

Cancer patients may be exposed to many different agents that can affect platelet function and coagulation during their medical or surgical treatment. In surgical patients, synthetic colloids are widely used as plasma substitutes because of their ability to increase and maintain circulating blood volume [6]. The effect of colloids on hemostasis may potentially be caused by the dilution effect and accompanying changes in blood viscosity and platelet count, an effect on the activity of blood coagulation factors and fibrinolysis, or an influence on platelet function [4]. Hartog et al. reported that the extent of coagulopathy depends on the pharmacokinetic properties of the HES molecules, such as MW or the DS of carbon atoms with hydroxyl moieties. HES 130/0.4 has a lower MW (130 kDa) and DS (0.4) and is suggested to affect coagulation to a lesser degree than HES 470/0.7 or HES 200/0.5. In their systemic review, 24 studies that assessed hemostasis by TEG were included and 19 of these studies confirmed that HES 130/0.4 leads to hypocoagulation. It was also mentioned that the effects on hemostasis may be clinically relevant, particularly at higher doses [11]. In a study by Ansari et al., parallel hypocoagulable results were reported with TEG in pregnant patients by using a mixture of lactate Ringer’s solution and HES (130/0.4) [12]. In another study including pregnant women at term, Turker et al. concluded that both HES and gelatin produce mild hypocoagulable changes, but thromboelastographic parameters remain within or just below the normal reference [13]. As with malignancy, pregnancy can cause hypercoagulability, and venous thromboembolism is also a leading cause of maternal mortality and morbidity.

The major result of the present study was that 6% HES 130/0.4 preparation caused significant hypocoagulable changes on the thromboelastographic profile of gynecologic cancer patients in vitro. HES negatively affected the speed of clot formation as evidenced by increasing of CFT and impaired clot strength as evidenced by shortening of MCF values in both INTEM and EXTEM assays. In other studies, different concentrations of HES 130/0.4 were included from 10% up to 40%. It was recommended that the maximum dose of HES 130/0.4 not exceed 15-20 mL/kg [14]. The volume of HES 130/0.4 (6%) used in our study was chosen to simulate the recommended blood replacement volume used clinically in perioperative settings. We used a modified rotation thromboelastogram (ROTEM®, Pentapharm) instead of conventional TEG because different aspects of the coagulation process can be detected earlier and better by ROTEM®. The most important benefits of ROTEM® technology include rapid availability of test results; less susceptibility to mechanical stress, movement, and vibration; and enhanced reproducibility [15]. The data are also continuous, digital, and retrievable for further calculations [16]. Our study identified the value of using thromboelastographic techniques in monitoring the effects of HES on the coagulation status of gynecologic cancer patients.

Our results suggest that HES could be a suitable synthetic colloid for plasma volume substitution during acute normovolemic hemodilution in the context of major abdominal surgery for gynecologic malignancy. As Hartog et al. discussed in their report, a hypocoagulatory effect as defined by a reduction in final clot strength may not be necessarily a bad thing in perioperative and postoperative periods, which are associated with hypercoagulable states [11]. However, it should not be forgotten that the interference of colloids with the system must be in an accurate balance since it may increase bleeding risk in much higher doses. If used correctly for the appropriate patient population intraoperatively, besides the advantage of decreasing exposure to allogenic blood transfusion, HES may avoid the risk of surgery-related thrombosis in patients with gynecologic malignancies.

However, the clinical relevance of this in vitro study has some limitations. Factors such as the physiological recruitment of platelets and coagulation factors in response to hemodilution or the effects of hypothermia and pharmacokinetics of HES are hard to study in vitro [12]. In vivo hemostasis studies are necessary for the assessment of hypercoagulability associated with intraoperative colloid regimen in cancer patients undergoing surgery.

In conclusion, 6% HES 130/0.4 solution caused significant hypocoagulable changes in the thromboelastographic profiles of gynecologic cancer patients in vitro. Thus, further investigations that correlate this hypocoagulability with the clinical picture are needed to determine if HES could be a more suitable synthetic colloid for plasma volume substitution during major abdominal surgery of gynecologic malignancies.

**Conflict of Interest Statement**

The authors of this paper have no conflicts of interest, including specific financial interests, relationships, and/ or affiliations relevant to the subject matter or materials included.

## Figures and Tables

**Table 1:**
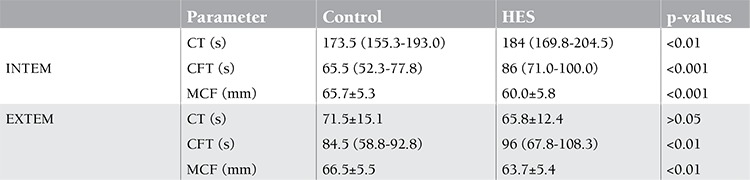
Values of ROTEM® parameters in the undiluted control and 10% HES diluted samples.
